# Single-Cell Multi-Omics in Type 2 Diabetes Mellitus: Revealing Cellular Heterogeneity and Mechanistic Insights

**DOI:** 10.3390/ijms262211005

**Published:** 2025-11-13

**Authors:** Yijie Wei, Feitong Hong, Sijia Xie, Xinwei Luo, Xiaolong Li, Fuying Dao, Kejun Deng, Hao Lin, Hao Lyu

**Affiliations:** 1 Department of Clinical Laboratory, Sichuan Clinical Research Center for Cancer, Sichuan Cancer Hospital & Institute, Sichuan Cancer Center, School of Life Science and Technology, University of Electronic Science and Technology of China, Chengdu 610054, China; 202421140421@std.uestc.edu.cn (Y.W.); 202421140509@std.uestc.edu.cn (F.H.); 202421140512@std.uestc.edu.cn (S.X.); 202421140508@std.uestc.edu.cn (X.L.); 202421140527@std.uestc.edu.cn (X.L.); dengkj@uestc.edu.cn (K.D.); 2 School of Biological Sciences, Nanyang Technological University, Singapore 639798, Singapore; fuying.dao@ntu.edu.sg

**Keywords:** type 2 diabetes mellitus, single-cell multi-omics, cellular heterogeneity, β-cell dysfunction, epigenomics

## Abstract

Type 2 diabetes mellitus (T2DM) is a prevalent and complex metabolic disorder characterized by insulin resistance, progressive β-cell dysfunction, and severe systemic complications. Advances in single-cell multi-omics—transcriptomics, chromatin accessibility profiling, and integrative analyses—have offered unprecedented insights into the cellular heterogeneity and regulatory networks of pancreatic islets. We highlight recent discoveries in islet cell heterogeneity and β-cell pathophysiology, with a particular focus on dysfunction and dedifferentiation. We further underscore the computational frameworks that enable these discoveries, spanning data preprocessing, multi-omics integration, and machine learning-driven analyses, which collectively enable the dissection of disease-relevant cell subpopulations and the reconstruction of developmental and regulatory trajectories. We also examine how impaired signaling within islets and chronic adipose inflammation contribute to T2DM pathogenesis. Finally, we discuss key challenges in clinical translation—including limited population diversity in single-cell atlases and the interpretability of computational models—and propose future directions toward precision diagnostics and therapeutic innovation in T2DM.

## 1. Introduction

Type 2 diabetes mellitus (T2DM) is a multifactorial metabolic disorder characterized by progressive β-cell dysfunction and systemic insulin resistance. Despite decades of research, its pathogenesis remains incompletely understood, partly due to the inherent cellular heterogeneity within metabolic tissues. Traditional bulk omics approaches, although capable of identifying global transcriptional changes in islets and adipose tissue, often obscure critical subpopulation-specific alterations. For instance, β-cell dedifferentiation, marked by loss of insulin (*INS*) expression and acquisition of progenitor markers, has been observed in only a subset of cells in human T2DM islets, a phenomenon masked in bulk RNA-seq studies [1]. Similarly, adipose tissue inflammation in obesity involves dynamic crosstalk between macrophages, adipocytes, and stromal cells, yet bulk analyses fail to resolve the spatial and temporal coordination of these interactions [2,3,4].

The advent of single-cell multi-omics technologies has revolutionized our capacity to dissect this heterogeneity in T2DM. By integrating transcriptomic, epigenomic, and proteomic data at single-cell resolution, these approaches have uncovered previously unrecognized cellular states and regulatory dynamics associated with disease progression. For example, single-cell RNA sequencing (scRNA-seq) of human pancreatic islets has identified distinct β-cell subpopulations (*INS*high and *INS*low) exhibiting stress-response pathways [5]. Similarly, single-cell ATAC-seq (scATAC-seq) has revealed T2DM-associated chromatin remodeling at regulatory regions of β-cell identity transcription factors, such as Hepatocyte Nuclear Factor 1 Alpha (HNF1A) [6]. Concurrent advances in computational tools, including batch correction algorithms (e.g., Harmony, Batch Balanced K-Nearest Neighbors (BBKNN)) and multi-omics integration frameworks (e.g., Seurat Weighted Nearest Neighbor (WNN), Multi-Omics Factor Analysis v2(MOFA+)), have further facilitated the systematic deconvolution and interpretation of these complex datasets.

Despite these advances, the rapid proliferation of single-cell studies has brought to light several critical methodological challenges. Technical noise, sparse data, and platform-specific biases complicate cross-study comparisons, while the biological interpretation of machine learning-derived cell states remains contentious. For example, clustering algorithms applied to β-cell transcriptomes often yield divergent subpopulation definitions depending on normalization strategies [7,8], raising questions about the robustness of inferred cellular trajectories. Moreover, while ligand-receptor interaction tools (e.g., CellChat, CellPhoneDB) have mapped altered crosstalk in T2DM islets [9,10], their predictions require rigorous experimental validation.

This review synthesizes recent advances in applying single-cell multi-omics to T2DM research. While previous reviews have largely cataloged biological insights gained from single-cell technologies, we uniquely focus on evaluating and contextualizing the computational frameworks that underpin these discoveries. We first discuss the application of data preprocessing and integration tools for resolving cellular heterogeneity in islet and adipose tissues and then explore how these methods have elucidated mechanisms of β-cell failure, including dedifferentiation and altered regulatory networks.

**Figure 1:**
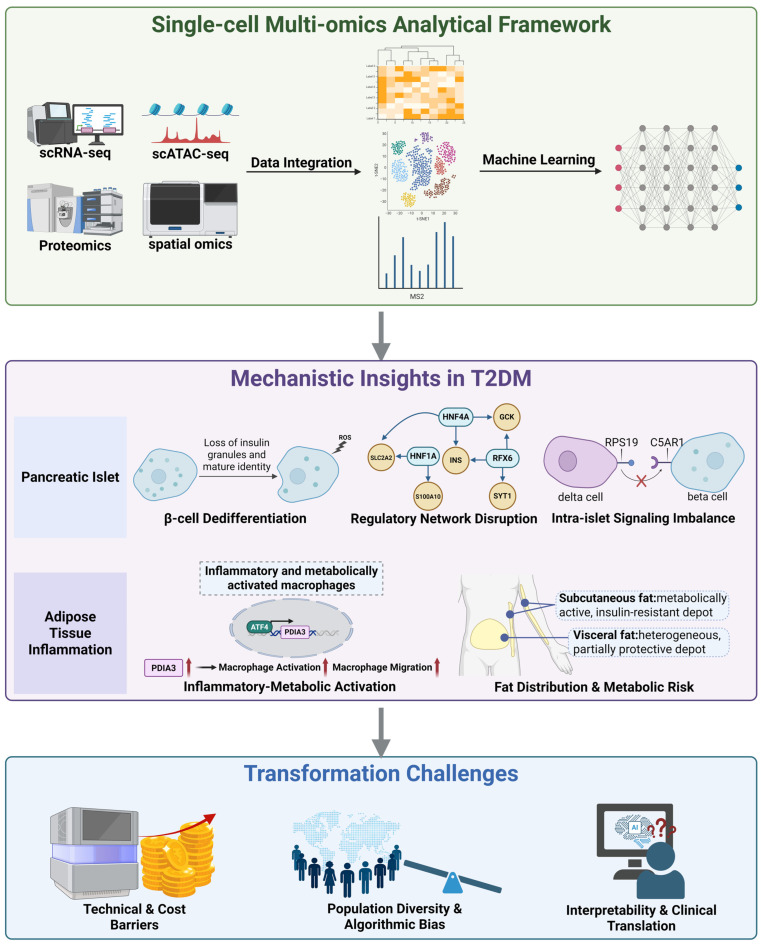
Integrated single-cell multi-omics framework reveals mechanistic and translational insights in T2DM. Single-cell multi-omics technologies, including scRNA-seq, scATAC-seq, and proteomics, enable data integration and machine learning based analysis to uncover key cellular and molecular mechanisms underlying type 2 diabetes. Mechanistic insights derived from these datasets reveal β-cell dedifferentiation, transcriptional network disruption (HNF1A, Hepatocyte Nuclear Factor 4 Alpha (HNF4A), Regulatory Factor X6 (RFX6)), and intra-islet signaling imbalance (Complement Component 5a Receptor 1 (C5AR1)-Ribosomal Protein S19 (RPS19)) within pancreatic islets, as well as inflammatory metabolic macrophage activation (Transcription Factor 4 (ATF4)-Protein Disulfide Isomerase Family A Member 3 (*PDIA3*)) and depot-specific differences between subcutaneous and visceral fat that shape insulin resistance and metabolic risk. Despite these advances, major challenges remain in translating such findings to clinical applications, including technical and cost barriers, population diversity and algorithmic bias, and gaps in model interpretability and clinical validation. Black arrows indicate workflow or regulatory direction; red arrows denote upregulation. Created in BioRender. Wei, Y. (2025) https://BioRender.com/ea1kpg4.

Unlike prior reviews that are either islet-centric or primarily methodological, this work provides a unified and clinically oriented perspective that explicitly links computational design to biological interpretability and reproducibility [11,12]. By integrating cross-tissue single-cell findings from pancreatic islets and adipose depots, we outline how molecular mechanisms across metabolic tissues converge to shape T2DM pathophysiology, and we compile a benchmark table summarizing the impact of computational frameworks on downstream biological inference to guide tool selection in diabetes studies. Finally, we address persistent translational challenges such as limited cohort diversity and model interpretability. By directly bridging algorithmic advances to disease mechanisms and clinical application, this review establishes a comprehensive roadmap from computational technique to therapeutic innovation in T2DM. To provide a conceptual overview, Figure 1 summarizes how single-cell multi-omics technologies connect data integration and computational modeling with biological discoveries and translational challenges, linking analytical advances to mechanistic understanding and clinical relevance.

## 2. Methods of Literature Search

To ensure comprehensive coverage, literature was retrieved from PubMed, Web of Science, and Google Scholar up to October 2025, using combinations of the keywords “single-cell”, “multi-omics”, “type 2 diabetes”, “β-cell”, and “adipose”. Priority was given to peer-reviewed original research and high-impact reviews published between 2015 and 2025 in journals such as Nature Metabolism, Cell Metabolism, and Nature Communications. Earlier foundational studies predating 2015 were also included when they provided essential conceptual or technical groundwork for single-cell or omics methodologies (for instance, early RNA-seq and β-cell physiology studies). Studies were selected if they (i) applied single-cell or multi-omics approaches to human or mammalian T2DM samples and (ii) contributed to mechanistic, computational, or translational understanding of disease pathophysiology.

## 3. Computational Frameworks for Decoding Cellular Heterogeneity in T2DM

### 3.1. Single Cell Insights into Cellular Heterogeneity

#### 3.1.1. Functional Variability in Pancreatic Islet Endocrine Cells

The heterogeneity of pancreatic islet cells refers to the significant functional, transcriptional, and epigenomic variability observed among cells within the same nominal type. Recent advancements in single-cell omics technologies have enabled high-resolution dissection of this heterogeneity.

For instance, single-cell chromatin accessibility profiling has uncovered multi-layered regulatory networks underlying the functional heterogeneity of islet endocrine cells [13]. In β-cells, distinct epigenetic programming stratifies cells into *INS*-high (high insulin-secreting) and *INS*-low (low-secreting) functional states: The *INS*-high subpopulation maintains robust hormone synthesis and secretion capacity through marked chromatin accessibility at the insulin–insulin-like growth factor 2 (*INS-IGF2*) promoter and enrichment of secretory pathway genes (e.g., chromogranin A (*CHGA*), secretogranin V (*SCG5*)). In contrast, *INS*-low β-cells exhibit transcriptional reprioritization toward endoplasmic reticulum (ER) stress pathways and pro-inflammatory signaling, showing remarkable concordance with the ER stress-associated β-sub.4 cluster identified through scRNA-seq [14]. Analogous state stratification occurs in α-cells, where *GCG*-high (high glucagon-secreting) subpopulations preserve glucagon secretion competence via a 3.67-fold increase in chromatin accessibility at the *GCG* promoter. Conversely, *GCG*-low α-cells adapt to stress microenvironments through Mitogen-Activated Protein Kinase (MAPK) pathway activation, illustrating conserved mechanisms of functional plasticity across islet endocrine cell types.

#### 3.1.2. Adipose Tissue as a Driver of Metabolic Inflammation

Beyond pancreatic islets, T2DM is also driven by inter-organ interactions, particularly involving adipose tissue. Obesity, a major risk factor for T2DM, is often accompanied by chronic low-grade inflammation in adipose tissue, which exacerbates systemic insulin resistance [15]. Adipose precursor cells (APCs), the predominant stromal cell population in adipose tissue, have now been identified as a highly heterogeneous group [16,17]. Single-cell analyses have revealed four distinct APC subpopulations in human visceral adipose tissue: Decay-Accelerating Factor-positive (*CD55^+^*), Cluster of Differentiation 9-positive and CD55-low (*CD9^+^CD55^low^*), Intercellular Adhesion Molecule 1-positive (*ICAM1^+^*), and Tissue Factor-positive (*CD142^+^*) [18].

Among these, the *CD9^+^CD55^low^* APCs subpopulation is significantly expanded in T2DM patients, with its abundance positively correlating with fasting blood glucose and glycated hemoglobin (HbA1c) levels. Transplantation of *CD9^+^CD55^low^* APCs from T2DM patients into mouse adipose tissue induces glucose intolerance and insulin resistance, while genetic knockout or pharmacological depletion of this subpopulation ameliorates obesity-associated metabolic dysfunction. Mechanistically, this subpopulation creates a pathogenic microenvironment by secreting bioactive factors such as midkine (MDK) and pigment epithelium-derived factor (PEDF), which promote adipocyte lipolysis, elevate serum free fatty acid and glycerol levels, and subsequently trigger hepatic lipid accumulation and enhanced gluconeogenesis.

#### 3.1.3. β-Cell Dedifferentiation and Developmental Reprogramming

At the islet level, progressive dysfunction in β-cells not only reflects phenotypic heterogeneity but also dedifferentiation, a process where mature β-cells lose their functional identity. This is typically characterized by reduced expression of insulin-secreting machinery and degranulation [19], i.e., loss or structural damage of insulin granules [20,21].

**Figure 2:**
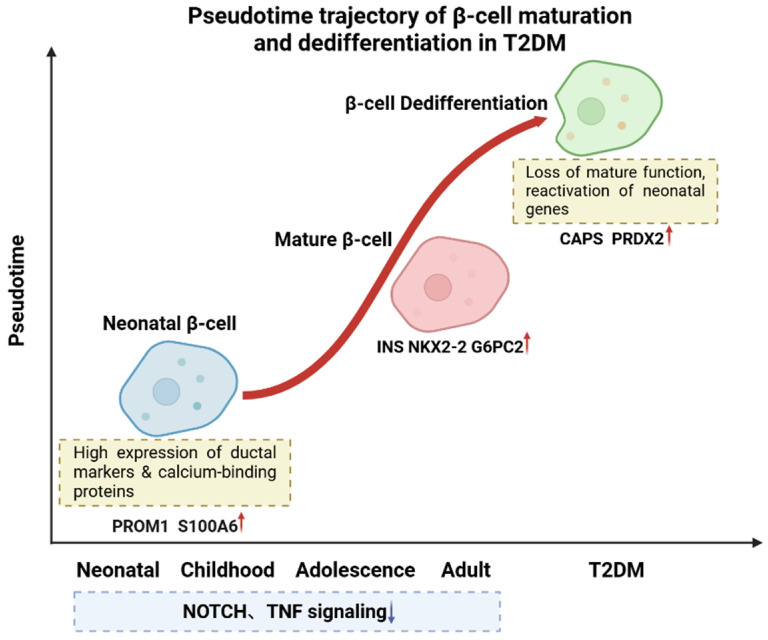
Pseudotime trajectory of β-cell maturation and dedifferentiation in T2DM. Developmental trajectory of β-cells progresses from neonatal to mature stages, with neonatal β-cells expressing genes such as *PROM1* and *S100A6* and mature β-cells expressing functional genes like *INS* and *G6PC2*. In T2DM, β-cells undergo dedifferentiation, reverting to an immature state and reactivating neonatal genes such as *CAPS* and *PRDX2*. During normal maturation, pathways like NOTCH and Tumor Necrosis Factor (TNF) signaling are gradually suppressed. Red arrows indicate upregulation; blue arrows indicate downregulation. Created in BioRender. Wei, Y. (2025) https://BioRender.com/j65al3o.

Using pseudotime analysis to track the transcriptomic dynamics of T2DM β-cells revealed that diabetic β-cells exhibit a pronounced reversal of developmental trajectories, characterized by the systematic reactivation of immature gene programs [22]. As illustrated in Figure 2, β-cells segregate into distinct clusters according to donor age (neonatal, early childhood, adolescence, and adulthood), with neonatal β-cells distinguishable from later developmental stages by their unique gene expression profile—marked by high expression of ductal cell markers such as Prominin-1 (*PROM1*) and Nuclear Factor I B (*NFIB*), as well as calcium-binding proteins like S100 Calcium-Binding Protein A11 (*S100A11*) and S100 Calcium-Binding Protein A6 (*S100A6*) [23]. Pseudotime trajectory analysis identified five key gene modules that drive the dynamic maturation process of β-cells from neonatal to adult stages, evidenced by the gradual silencing of development-associated pathways and the sustained activation of β-cell functional genes (e.g., NK2 Homeobox 2 (*NKX2-2*) and Glucose-6-Phosphatase Catalytic Subunit 2 (*G6PC2*)). This analysis indicates that the transcriptomic profile of T2DM β-cells deviates from the normal maturation trajectory, reverting toward an immature neonatal and early childhood state, as exemplified by the expression levels of characteristic genes such as Calcyphosine (*CAPS*) and Peroxiredoxin 2 (*PRDX2*)—antioxidant and stress response genes—that are restored to neonatal levels.

### 3.2. Multi-Omics Approaches in T2DM Mechanism Discovery

Advancements in single-cell and multi-omics technologies have revolutionized our capacity to interrogate the cellular and molecular complexity underlying T2DM. By integrating innovations in transcriptomics, epigenomics, and proteomics with cutting-edge computational tools, researchers are now able to uncover previously inaccessible aspects of disease etiology, progression, and heterogeneity.

ScRNA-seq has been instrumental in mapping the diverse cellular states and transitions within diabetic tissues [24]. Unlike bulk RNA-seq, which averages gene expression across thousands of cells, scRNA-seq resolves transcriptional profiles at the individual cell level, revealing rare subpopulations and dynamic processes such as immune cell activation and β-cell dedifferentiation [25,26]. Dimensionality reduction (e.g., Uniform Manifold Approximation and Projection (UMAP), t-distributed Stochastic Neighbor Embedding (t-SNE)) and graph-based clustering (e.g., Louvain, Leiden) algorithms enable the precise identification of functionally distinct cell types [27,28]. For example, scRNA-seq has uncovered eight peripheral T cell subtypes with divergent roles in T2DM progression, including regulatory CD4^+^ T cells and cytotoxic CD8^+^ T cells [29]. Pseudotime analysis tools such as Monocle and Slingshot further reconstruct developmental or disease trajectories, offering insights into how cellular states evolve during metabolic dysfunction [30,31].

Single-cell chromatin accessibility profiling complements scRNA-seq by revealing the epigenetic landscape that governs transcriptional activity [32]. scATAC-seq identifies open chromatin regions, transcription factor binding sites, and regulatory elements at single-cell resolution [33]. In the context of T2DM, this enables the inference of cell-type-specific gene regulatory networks and the discovery of epigenetic alterations associated with metabolic stress or inflammation [34]. Integrative frameworks, such as ArchR and Signac, combine scATAC-seq with scRNA-seq to jointly model gene expression and regulatory control, providing a mechanistic view of how β-cell identity is maintained or lost under diabetic conditions [35,36].

Proteomics, particularly at the single-cell level or through spatially resolved platforms, offers another layer of insight by quantifying functional protein expression and post-translational modifications [37]. Mass spectrometry-based and affinity-based approaches have identified protein-level signatures of insulin resistance, islet inflammation, and immune dysregulation [38,39]. Recent developments in single-cell proteomics, such as Cytometry by Time-of-Flight (CyTOF) and CITE-seq, allow for simultaneous quantification of surface proteins and transcriptomes in the same cell, bridging the gap between mRNA abundance and functional protein output [40,41,42]. This is critical for understanding phenomena such as the disconnect between transcript levels and cytokine activity in T2DM immune cells.

Together, these multi-omics technologies—enabled and interpreted through sophisticated computational methods—constitute a new paradigm in diabetes research. They shift the focus from bulk-level observations to cell-specific, mechanistic insights, allowing researchers to characterize not only what changes in T2DM, but also how and in which cell types these changes occur. This integrated, systems-level approach is essential for the identification of novel biomarkers, therapeutic targets, and personalized treatment strategies.

## 4. Computational Toolbox Support Multi-Omics Analysis in T2DM

### 4.1. Preprocessing Strategies Improve Single-Cell Data Quality

Single-cell omics techniques such as scRNA-seq provide unprecedented resolution for studying cellular heterogeneity. However, the inherent high noise, sparsity, and technical biases in these data present significant challenges for analysis. These biases include differences in sequencing depth, gene-length and GC-content preferences, systematic errors from batch or platform variations, and the “dropout” phenomenon where lowly expressed genes are undetected due to technical limitations [43,44].

To mitigate these issues, data preprocessing begins with normalization, which aims to correct for differences in sequencing depth and distributional biases, thereby ensuring comparability of expression levels across cells. While simple scaling methods such as counts per million (CPM) or transcripts per million (TPM) are commonly used, they may overlook the complexity and variability in expression distributions among cells. As a result, more robust statistical approaches have been developed. For example, DESeq2’s median ratio method assumes most genes remain unchanged across conditions and calculates scaling factors accordingly [45]. The Scran package further improves normalization accuracy in sparse datasets by pooling cells with similar expression profiles to estimate size factors more reliably [46]. Following normalization, logarithmic transformation (e.g., log(CPM + 1)) or regularized negative binomial models such as SCTransform can be applied to stabilize the variance–mean relationship in gene expression [47], thereby enhancing the reliability of downstream analyses such as clustering and differential expression. By applying such optimized normalization pipelines, recent single-cell analyses have successfully discerned subtle β-cell state changes under metabolic stress that were previously difficult to resolve [48].

A common artifact in scRNA-seq data is the dropout phenomenon, where transcripts of low-abundance genes are missed due to technical limitations, resulting in an excess of zero counts. This sparsity can hinder the detection of subtle biological signals. To address this, several imputations and denoising methods have been developed. For instance, Clustering through Imputation and Dimensionality Reduction (CIDR) mitigates dropout effects by implicitly imputing missing gene expression values [49]. Deep Count Autoencoder (DCA) leverages deep neural networks to learn a latent representation of the data and reconstructs denoised gene expression matrices by modeling the negative binomial noise structure inherent in scRNA-seq data [50]. Another widely used method, Markov Affinity-based Graph Imputation of Cells (MAGIC), constructs a graph-based representation of cellular similarity and uses diffusion processes to propagate gene expression information across neighboring cells, effectively smoothing the data and recovering underlying gene–gene relationships [51]. Although these approaches differ in methodology, they share a common goal: to mitigate dropout noise and restore a meaningful signal for accurate biological interpretation.

Batch effects represent an inevitable source of technical noise in single-cell omics data analysis, primarily arising from non-biological factors such as variations in experimental conditions, sample processing times, differences in sequencing platforms, or changes in operators [52,53,54,55]. This technical variability can obscure genuine biological differences, leading to unreliable comparisons across samples or studies [56]. For example, in scRNA-seq, differences in cell capture efficiency, library preparation protocols, or sequencing depth across batches can significantly affect the distribution of the gene expression matrix [54]. Given the persistence and severity of batch effects, a number of integration tools have been developed specifically for batch correction. Harmony, for example, applies a linear embedding model combined with iterative soft clustering to dynamically align cell distributions across batches [57]. It starts from an initial Principal Component Analysis (PCA) reduction and refines cell embeddings over iterations, effectively removing batch-driven differences while preserving biological structure. Its strengths lie in scalability to millions of cells and sensitivity to rare populations, making it particularly useful for multi-platform or multi-batch integration tasks [58]. Seurat’s integration framework, including Canonical Correlation Analysis (CCA) and SCTransform-based anchor matching [32], identifies “anchor cell pairs” across batches and uses their expression differences to adjust the global gene expression space. While this approach excels at maintaining cell type-specific signals [59], it is memory-intensive and sensitive to parameter tuning, which can lead to overcorrection if not carefully calibrated [54]. To evaluate the effectiveness of preprocessing, including batch correction, it is recommended to visualize data using dimensionality reduction techniques such as UMAP or PCA, assess the distribution of highly variable genes, and verify that clustering results align with known biological identities. Recent studies also emphasize the need to balance computational efficiency, scalability, and reproducibility, especially when integrating multi-omics datasets involving multiple modalities [60]. By harmonizing datasets across platforms and studies, such integration techniques not only correct batch bias but also reveal biological insights. For instance, an integrated single-cell atlas defined 19 distinct β-cell states and showed that certain immature β-cell subpopulations upregulate innate immune response genes, reflecting crosstalk between islet cells and the immune system in T2DM [61].

A summary table (Table 1) compares representative algorithms for normalization, dropout recovery, and batch-effect correction, outlining their core principles, limitations, and potential impacts on biological interpretation to facilitate informed method selection in single-cell studies.

In summary, normalization, scaling, and batch correction are foundational steps in single-cell omics data preprocessing. Their careful and context-aware application is critical to minimizing technical noise while preserving the biological signals essential for accurate downstream interpretation.

### 4.2. Integration Techniques Enable Cross-Modal Data Fusion

**Figure 3:**
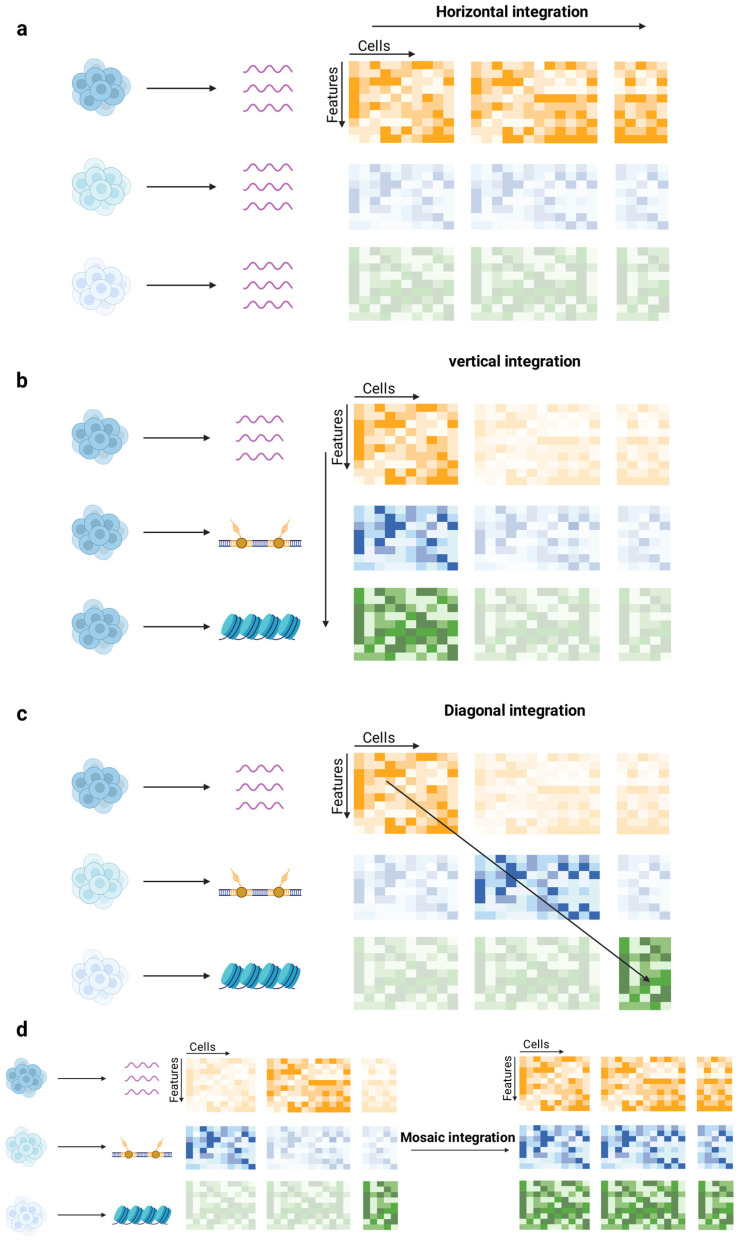
Strategies for single-cell multi-omics data integration. (**a**) Horizontal integration: Aligns independently measured datasets based on shared features to identify conserved cell types. (**b**) Vertical integration: Combines non-overlapping omics data from the same cells, preserving cross-modal correspondence. (**c**) Diagonal integration: Projects distinct modalities without shared anchors into a shared latent space for cross-modal analysis. (**d**) Mosaic integration: Unifies heterogeneous datasets with missing modalities into a coherent multimodal system, leveraging both shared and non-shared features. Different colors represent result matrices of distinct omics modalities, and transparent matrices indicate missing or unmeasured modalities. Created in BioRender. Wei, Y. (2025) https://BioRender.com/gddpggm.

Data integration is an indispensable step in single-cell multi-omics studies, with the primary goal of effectively combining data from different sources or modalities [73]. Based on the characteristics of the data and the integration objectives, data integration can be categorized into four main strategies: horizontal integration, vertical integration, diagonal integration, and mosaic integration [74,75] (Figure 3).

#### 4.2.1. Horizontal Integration Strategy

Horizontal integration aims to combine independently measured cell populations based on shared features. This approach is typically employed to integrate data from different batches measured with the same technology or to combine results from different technical assays of the same molecular layer [74]. It is particularly effective at mitigating batch effects and experimental variability, enabling the detection of conserved cell types and rare populations. However, horizontal integration relies on high-quality shared features, and noisy or sparse features can hinder accurate alignment, potentially leading to artificial clustering or loss of biological variation.

For instance, Stuart et al. developed the CCA method in Seurat v3 [32], which aligns datasets by identifying shared sources of variation using highly variable genes as anchors. They demonstrated its utility in integrating scRNA-seq datasets from different tissues and identifying conserved cell types across organs, while also uncovering rare populations obscured by batch effects.

Another example is Harmony, introduced by Korsunsky et al. [57], which uses a soft clustering approach to iteratively align datasets in a shared low-dimensional space. Harmony was shown to integrate multi-donor scRNA-seq datasets, successfully correcting for inter-individual variation and identifying shared immune cell subsets across donors.

In addition to these methods, Welch et al. developed Linked Inference of Genomic Experimental Relationships (LIGER) [76], which employs non-negative matrix factorization to separate shared and dataset-specific factors. They applied LIGER to integrate healthy and diseased tissue scRNA-seq data, uncovering both conserved transcriptional programs and condition-specific gene expression signatures.

Beyond computational performance, horizontal integration aligns independently profiled islet datasets to recover conserved disease-relevant cell states, thereby increasing power to detect regulatory programs that are reproducibly perturbed in T2DM across donors and cohorts. Using cross-dataset alignment, single-cell chromatin accessibility maps have defined β-cell-state-specific regulatory elements that concentrate T2DM risk variants, directly linking integrated signals to mechanisms of insulin-secretory failure [13]. Collectively, these integrative efforts demonstrate that horizontal integration not only corrects batch-related variability but also deepens biological interpretation, allowing disease-linked regulatory mechanisms to be compared and validated across human cohorts.

Integration of large-scale single-cell transcriptomic datasets can indeed reveal previously unrecognized β-cell states in diabetes. For example, Hrovatin et al. integrated > 300,000 islet cells from multiple mouse models into a cross-condition atlas, uncovering novel transitional β-cell states emerging during T2DM progression [61]. This integrated atlas highlighted dynamic β-cell dedifferentiation and shared stress-response pathways across disease models.

#### 4.2.2. Vertical Integration Strategy

Vertical integration anchors on individual cells to integrate non-overlapping omics data derived from the same cell [75]. This approach is suitable for technologies that simultaneously measure multiple omics layers in single cells. For example, in human β-cells, single-cell multiome analysis that jointly models transcriptomes and chromatin accessibility identified HNF1A as a principal driver of intra-donor heterogeneity and β-cell dysfunction in T2D, illustrating how within-cell RNA–ATAC coupling pinpoints causal regulators rather than correlative markers [6]. Its main advantage lies in the ability to preserve the intrinsic correspondence between different omics layers within the same cell, thus allowing for more accurate modeling of regulatory relationships and cell states. However, vertical integration often requires complex experimental protocols and is limited by the availability of multi-modal single-cell technologies, which may result in sparse or noisy measurements for certain modalities.

For instance, Stuart et al. developed the WNN approach, implemented in Seurat v4 [77], which integrates multi-omics data by calculating the relative utility of each modality for capturing cellular heterogeneity. Specifically, WNN identifies influential feature pairs across modalities, such as chromatin accessibility from ATAC-seq and gene expression from RNA-seq, and constructs a unified neighbor graph that reflects both data modalities. The authors showed that WNN can robustly resolve cell types in single-cell multiome datasets and accurately map new query datasets onto reference atlases using shared features (e.g., marker genes or regulatory elements). This capability enables effective cross-sample comparisons and the discovery of novel cell states.

Another example is a probabilistic framework, Total Variational Inference (totalVI), introduced by Gayoso et al. [78], which integrates paired gene and protein measurements such as those generated by CITE-seq, a technology that combines RNA-seq with surface protein profiling. This method employs a deep generative model that accounts for technical noise, batch effects, and sparsity in the data while simultaneously modeling the relationships between RNA and protein expression. Gayoso et al. showed that totalVI could uncover coordinated regulation between transcriptomic and proteomic layers, such as the concordance between cytokine receptor expression at the transcript and protein levels. Moreover, totalVI enabled the identification of distinct immune cell subsets in complex tissues, such as tumor microenvironments, by resolving subtle transcript-protein mismatches that are often masked by technical noise in raw multi-omics data.

#### 4.2.3. Diagonal Integration Strategy

The diagonal integration strategy holds particular promise for T2DM research because it allows the alignment of independently profiled, heterogeneous modalities even when no shared cells or features exist. By projecting each modality into a shared latent space via methods such as autoencoders or probabilistic couplings, this approach enables cross-modal biomarker discovery and regulatory program reconstruction in the absence of direct anchors [79]. In this latent space, one can analyze correlations between molecular layers, identify multimodal biomarkers, and enable data exchange between different modalities—although this may come at the expense of some single-cell resolution [74].

ScConfluence, a diagonal integration method, was proposed to combine uncoupled autoencoders and regularized Inverse Optimal Transport (rIOT) [79]. This method reduces the dimensionality of the original data into a shared latent space using autoencoders, while rIOT ensures alignment of cell embeddings across modalities by leveraging weakly connected features. Unlike conventional approaches, scConfluence independently processes the complete set of original features while utilizing only the connected features for alignment, thus avoiding the loss of biological information typically caused by prior modality conversion.

Chen et al. recently proposed MaxFuse, a cross-modal data integration algorithm designed to align weakly linked multimodal datasets [80]. MaxFuse takes both all-feature and linked-feature matrices from two modalities as input. It begins by constructing fuzzy nearest-neighbor graphs to smooth linked features and performs initial cell matching using a linear assignment algorithm. Then, it employs CCA to learn cross-modal joint embeddings, iteratively refining cell matches through smoothing and matching updates. High-quality matched pairs are filtered and propagated, ultimately generating a unified embedding space for all cells. This approach is particularly notable for its modality agnosticism, high robustness, and computational efficiency, making it well-suited for multimodal data integration in challenging scenarios.

Although applications in T2DM remain preliminary, diagonal integration provides a promising avenue for uncovering cross-modal regulatory programs and disease-associated cellular states that single-modality analyses cannot resolve. Expanding its use in islet and metabolic tissue studies could reveal inter-layer mechanisms and advance mechanistic understanding of diabetes pathogenesis.

#### 4.2.4. Mosaic Integration

Mosaic integration addresses the surge of single-cell multi-omics datasets that combine heterogeneous modality subsets across studies, thereby creating a “mosaic” pattern of missing data and modalities [74]. Mosaic integration tackles these challenges by combining datasets in which different modalities are measured on distinct subsets of cells or under varying experimental conditions. Its main objectives are to project multimodal data into a shared latent space using both shared and unshared features and to enable transfer learning from a reference dataset to another that contains only a subset of modalities [81]. This allows the inference of missing molecular layers at reduced experimental cost, effectively mitigating data sparsity through computational imputation [82]. Beyond mere algorithmic scalability, this strategy opens new avenues in disease biology by enabling inference of missing molecular layers and thereby reconstructing more complete multimodal profiles of individual cells or cell-states—an advance especially pertinent to complex disorders such as T2DM, where multiple regulatory layers (transcriptome, epigenome, proteome) interplay [83].

Several advanced methods have been developed to address mosaic integration. Kriebel et al. proposed Unshared Integrative Non-negative Matrix Factorization (UINMF), a non-negative matrix factorization algorithm designed to integrate single-cell multi-omic datasets with both shared and unshared features [84]. By leveraging common features for alignment and unique features for modality-specific optimization, UINMF enables the integration of mismatched datasets, such as scRNA-seq with spatial transcriptomic data or cross-species datasets. It learns latent metagenes that capture shared biological signals while preserving unique variations, enhancing integration accuracy over methods relying only on shared features.

Building on this progress, Ghazanfar et al. proposed StabMap [85], a prominent approach that constructs a Mosaic Data Topology (MDT), a weighted network where nodes represent datasets and edges reflect shared features [86]. Reference datasets are embedded into low-dimensional coordinates using PCA or discriminant analysis, while non-reference datasets are projected onto these coordinates via shortest-path traversal on the MDT. StabMap preserves cell–cell relationships, enabling batch correction and cell type prediction, even for datasets with few or no shared features.

### 4.3. Machine Learning Guides Cell State Identification and Annotation

With the rapid advancement of single-cell transcriptomics and epigenomics technologies, single-cell resolution multi-omics data provide unprecedented opportunities for disease mechanism analysis and precision medicine. However, the high dimensionality, sparsity, heterogeneity, and technical noise of these data pose significant challenges to traditional analytical methods. The integration of machine learning into single-cell omics is accelerating the field towards greater precision and efficiency [87,88,89].

Machine learning algorithms can enhance signal recovery and feature reconstruction for low-abundance cell populations. One study developed a deep learning model based on the U-shaped Convolutional Neural Network (U-Net) [90] architecture to address the challenge of predicting open chromatin peaks in pancreatic rare cell populations (δ cells, accounting for <5%) from single-cell ATAC-Seq data [91]. Inspired by image super-resolution techniques, the model takes sparse, low-depth single-cell data as input and predicts high-resolution chromatin peak signals. The results showed that this model effectively restored peak signals from sparse data and outperformed the traditional Model-based Analysis of ChIP-Seq v2 (MACS2) method significantly. Moreover, the model maintained robust performance even with a small number of input cells (5–200 cells), providing an efficient tool for studying the function of rare cell populations.

In addition, machine learning also enables the integration of gene regulatory mechanisms and interaction networks to identify key regulators of cell state transitions. A recent study proposed a mechanism-driven deep neural network model called regX, specifically designed to analyze the regulatory mechanisms underlying pancreatic β-cell state transitions in T2DM [92]. By integrating gene regulatory networks and interactions, regX constructs a hierarchical structure incorporating gene subnetworks and graph neural networks (GNNs). Using T2DM datasets, regX identified key transcription factors such as GLIS Family Zinc Finger 3 (GLIS3) and RAR-Related Orphan Receptor Alpha (RORA) through virtual perturbation experiments, revealing their roles in driving disease progression by regulating β-cell apoptosis and insulin secretion.

Moreover, machine learning significantly improves the precision of cellular heterogeneity analysis and enables the accurate identification of cell subpopulations. One study trained an XGBoost classifier on single-cell chromatin accessibility data and successfully identified two β-cell subtypes with distinct functional and transcriptional characteristics through leave-one-out cross-validation—β-1 and β-2 [93]. Notably, the proportion of β-2 cells increased significantly during T2DM progression, with chromatin accessibility regions enriched in T2DM risk variants, closely associated with declining insulin secretion capacity.

In summary, machine learning is revolutionizing single-cell omics by enabling precise signal restoration, identification of disease-relevant regulators, and discovery of functionally distinct cell subpopulations. These approaches not only overcome technical limitations in single-cell data analysis but also provide mechanistic insights and translational potential for understanding complex diseases such as T2DM [94].

## 5. Multi-Omics Insights Clarify the Mechanisms of β-Cell Failure

### 5.1. Dissection and Regulatory Mechanisms of β-Cell Differentiation Trajectories

**Figure 4:**
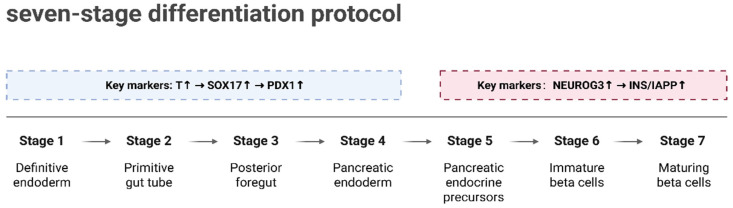
Seven-stage differentiation protocol for β-like cells. Differentiation is divided into seven stages (S1–S7). Early stages (S1–S4) involve the formation of pancreatic progenitor cells through sequential activation of embryonic markers (T, SOX17, PDX1). Later stages (S5–S7) lead to the functional maturation of β-like cells, with endocrine progenitor markers (NEUROG3, INS, IAPP) driving the process. Created in BioRender. Wei, Y. (2025) https://BioRender.com/bye7zri.

A major goal in diabetes treatment is the generation of large numbers of functional, transplantable β-cells from patient-derived pluripotent stem cells. Over the past decade, several in vitro protocols have been developed to differentiate human embryonic stem cells (hESCs) into pancreatic progenitors and further into functional stem cell-derived β-like cells (SC-β-cells) [85,95,96,97]. The application of single-cell multi-omics technologies has provided a new perspective for deciphering the molecular regulatory mechanisms underlying β-cell differentiation. For instance, Weng et al. [98] constructed a lineage tree comprising 95,308 single-cell transcriptomes, systematically revealing the entire differentiation trajectory from hESCs to β-like cells. The differentiation process was divided into multiple stages (S1–S7), as shown in Figure 4. In the early stages (S1–S4), the sequential activation of key embryonic markers (such as Brachyury (*T*), SRY-Box Transcription Factor 17 (*SOX17*), and Pancreatic and Duodenal Homeobox 1 (*PDX1*)) drove the formation of ref. [98] pancreatic progenitor cells; in the later stages (S5–S7), the expression of endocrine progenitor-specific genes (such as Neurogenin 3 (NEUROG3), INS, and Islet Amyloid Polypeptide (IAPP)) laid the foundation for the functional maturation of β-like cells. These findings provide critical insights into the multi-stage gene expression patterns during differentiation.

At the initiation of stem cell differentiation toward β-cells, the coordinated action of multiple transcription factors guides cell fate decisions [99]. Transcription factors such as NEUROG3 play a crucial role in inducing the endocrine lineage [100,101], and their dynamic expression levels influence the subsequent differentiation trajectory. Trajectory analysis based on chromatin accessibility further confirmed that early endocrine progenitors with high NEUROG3 expression constitute the common precursor for all endocrine cell lineages [102]. As differentiation progresses, a series of transcription factors (such as Paired Box 4 (PAX4) and Pancreatic and Duodenal Homeobox 1 (PDX1)) interact with cis-regulatory elements on chromatin through hierarchical regulatory networks [103,104], ultimately determining the differentiation of specific islet cell types.

During β-cell differentiation, the multimodal transcriptional regulatory features at different developmental stages have also been elucidated. Some genes are repeatedly activated across various stages of differentiation, whereas the corresponding enhancers usually display a single activation pattern [98]. For example, the stage-specific enhancer of the diabetes-associated gene Transcription Factor 7 Like 2 (TCF7L2) drives its biphasic expression pattern during β-cell differentiation—a pattern that plays a key role in β-cell fate determination and functional maturation. In addition, differences in the activation of signaling pathways significantly affect differentiation efficiency and maturity. For instance, compared with primary β-cells, SC-β-cells exhibit lower activation of insulin secretion-related pathways and higher activity in amino acid metabolism pathways [105], resembling the characteristics of immature β-cells in neonatal mammals [106]. Insufficient activity of pathway-related transcription factors (such as Signal Transducer and Activator of Transcription 3 (STAT3) and Aryl Hydrocarbon Receptor Nuclear Translocator Like 2 (ARNTL2)) [106,107] may be one reason for the limited differentiation efficiency of SC-β-cells.

Although current differentiation protocols can generate functional β-like cells, there remains room for improvement in both efficiency and maturity [108,109]. Studies have indicated that low expression of certain key transcription factors (such as MAF BZIP Transcription Factor A (MAFA)) limits the functional maturation of SC-β-cells. Additionally, the inclusion of non-endocrine cells remains a challenge [110]. These off-target cells, comprising 2–10% of the population, have been linked to cyst formation and graft enlargement in rodent models, highlighting the need to minimize their presence for safe clinical applications.

### 5.2. Dysregulation of Gene Regulatory Networks

In recent years, single-cell omics studies have revealed key hub genes within the gene regulatory network of T2DM. For example, RFX6 has been identified by multiple studies as a crucial driver of early β-cell dysfunction in T2DM. By integrating single-cell transcriptomics, ATAC-seq, and spatial proteomics data, researchers have found that RFX6 regulates the expression of exocytosis-related genes (such as ion channel genes involved in the insulin secretion pathway), thereby influencing β-cell function [111]. Its downregulation is directly associated with insulin secretion defects in T2DM patients. Another study utilized weighted gene co-expression network analysis (WGCNA) to construct gene co-expression modules for β-cells, α-cells, and the whole islet. It found that the β-cell module β01 was significantly positively correlated with insulin secretion function and was highly enriched for T2DM GWAS signals and RFX6 binding motifs [112].

In the islet microenvironment of T2DM, interactions between metabolic pathways and the immune system significantly impact gene regulatory networks. PhenoCycler-Fusion spatial single-cell proteomic technology has revealed that in T2DM patients, islet vasculature is reduced in size, the distance between α/β-cells and blood vessels increases, and CD3^+^ T-cell infiltration is elevated [111]. These spatial interaction changes may activate β-cell stress response pathways (such as the Nuclear Factor kappa-light-chain-enhancer of activated B cells (NF-κB) and c-Jun N-terminal Kinase (JNK) signaling pathways) through hypoxia or inflammatory signals, exacerbating insulin resistance. Additionally, abnormal spatial distribution of macrophages and T cells further disrupts islet homeostasis through cytokine signaling involving IL-1β and TNF-α.

Single-cell epigenomic studies have uncovered widespread abnormalities in chromatin accessibility and transcription factor activity in T2DM. For instance, in β-cells of T2DM patients, the Hepatocyte Nuclear Factor (HNF) transcription factor family exhibits dysregulated regulatory networks, leading to the misexpression of lipid metabolism and insulin synthesis-related genes such as *INS* and Glucokinase (*GCK*) [93]. Additionally, aberrant DNA methylation, such as hypermethylation of the Forkhead Box O1 (*FOXO1*) promoter, suppresses the insulin signaling pathway, while imbalances in histone modifications affect the activation of stress response genes. These epigenetic changes may be triggered by metabolic stress or chronic inflammatory signals.

### 5.3. Signaling Communication Between Tissues

Cell-to-cell communication plays a critical role in tissue development, regeneration, and function, and its disruption can lead to diseases and developmental abnormalities [113]. The revolution of single-cell genomics has provided unprecedented insights into cell identity and opened new avenues for dissecting the complex interactions within tissue niches.

In the islets, communication among cells is coordinated through mechanisms such as electrical coupling via gap junctions, direct cell-to-cell contact, and paracrine interactions, all of which are central to maintaining the overall function of the islet and blood glucose homeostasis [114,115]. Within the islet, β-cells regulate the secretion of glucagon by α-cells through the secretion of insulin [116], while the somatostatin secreted by δ cells exerts an inhibitory effect on both β and α-cells [117]. The comparison between intact islet cell functions and those of dispersed cells highlights the critical role of intra-islet communication. α, β, and δ cells depend on the islet microenvironment to function normally and lose their typical characteristics when isolated [118]. Furthermore, it is well established that cell-to-cell communication within the islet is not only crucial for normal hormone secretion but that defects in such communication can lead to aberrant hormone release, potentially triggering or exacerbating the progression of diabetes [119].

**Figure 5:**
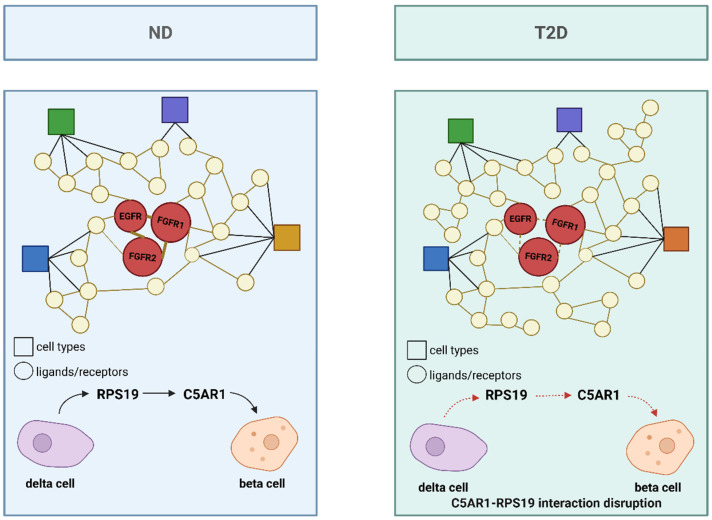
Islet interactome in ND and T2DM states. The interactome compares ligand–receptor interactions in ND and T2DM islets. In ND islets, interactions such as RPS19-C5AR1 between β and δ cells are maintained, contributing to islet homeostasis. In T2DM islets, the number of ligand–receptor interactions increases, but the C5AR1-RPS19 interaction is disrupted, potentially leading to reduced insulin secretion and increased apoptosis. Circles represent ligands/receptors, and squares represent cell types. Different colors represent distinct cell types. Solid lines indicate preserved or strengthened ligand–receptor interactions, whereas dashed lines represent weakened or disrupted interactions. Created in BioRender. Wei, Y. (2025) https://BioRender.com/o24sy55.

In studies of cell communication in T2DM using single-cell omics, the CellPhoneDB framework has been applied to analyze single-cell datasets from non-diabetic (ND) and T2DM donors, thereby constructing an islet interactome (Figure 5) [120]. CellPhoneDB is a bioinformatics toolkit designed to infer cell–cell communication by integrating a curated repository of bona fide ligand–receptor interactions with a series of computational and statistical methods, allowing for a deep exploration of intercellular signaling mechanisms within single-cell genomic data [10]. This tool accurately captures the multimeric nature of molecular complexes and faithfully represents the biology of cell communication. Studies have found that in ND islets, interactions such as INS–insulin receptor (INSR), glucagon (GCG)–glucagon receptor (GCGR), and somatostatin (SST)–somatostatin receptor (SSTR) among β-cells play key roles in maintaining islet homeostasis. However, in T2DM islets, the number of ligand–receptor interactions increases significantly (rising from 9707 to 10,787), and notable changes occur in β-cell-associated interactions, such as the loss of the C5AR1-RPS19 interaction between β and δ cells, which has been linked to reduced insulin secretion and increased cell apoptosis [121].

Another study utilized the CellChat tool to analyze the immune cell communication network in peripheral blood mononuclear cells (PBMCs) from T2DM patients and healthy controls (HCs) [29]. CellChat is capable of inferring and analyzing intercellular communication networks from scRNA-seq data as well as spatial transcriptomics data [9]. It employs a simplified mass action-based model to quantify the probability of signaling communication between two cell groups, integrating core interactions between ligands and receptors with multimeric structures while taking into account the regulation by cofactors. Compared to earlier tools, CellChat offers improved sensitivity and interpretability in signaling inference, though it remains limited by reliance on known ligand–receptor databases. The study found that the signaling intensity in T2DM subtypes A, B, and C, which were classified based on metabolic diversity within T cell subpopulations, was significantly higher than in the HC group (subtype D), with the number of interactions increasing by 17–20%. Among these, subtype B exhibited the strongest communication activity, involving key pathways such as Cluster of Differentiation 30 (CD30), Cluster of Differentiation 48 (CD48), Transforming Growth Factor Beta (TGF-β), and Interferon Gamma (IFN-γ), suggesting its central role in immune regulation and inflammatory responses.

## 6. Adipose Inflammation Shapes the Pathophysiology of T2DM

Chronic low-grade inflammation of adipose tissue is one of the key features of T2DM. In patients with T2DM, the inflammatory state of adipose tissue is closely associated with insulin resistance and glycemic control. Specifically, this is characterized by the infiltration of macrophages and other immune cell populations into the adipose tissue, along with a shift of leukocytes toward a more pro-inflammatory phenotype [122]. In normal adipose tissue, macrophages are predominantly anti-inflammatory M2-type cells that help maintain tissue homeostasis. However, in the context of obesity, the number of infiltrating M1-type macrophages increases significantly, a change that is primarily driven by obesity-induced alterations in the adipose tissue microenvironment [2].

**Figure 6:**
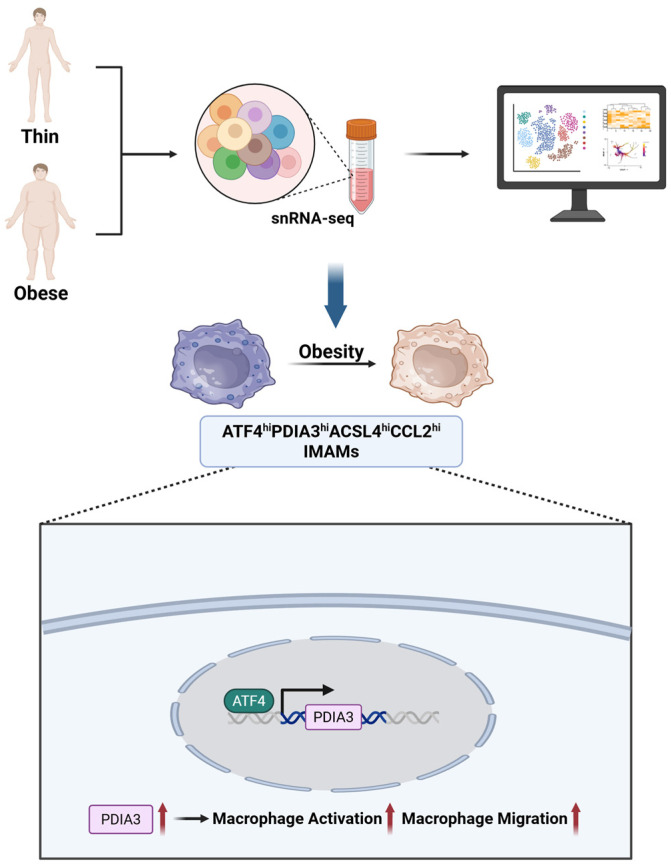
Identification of IMAMs in obesity-associated adipose tissue. snRNA-seq reveals a distinct macrophage subpopulation (*ATF4^hi^PDIA3^hi^ACSL4^hi^CCL2^hi^* IMAMs) enriched in obese visceral fat, with activation of pro-inflammatory and metabolic dysregulation pathways. The bottom panel shows ATF4-PDIA3-driven macrophage activation and migration. Created in BioRender. Wei, Y. (2025) https://BioRender.com/sr89vhu.

Nevertheless, Blériot et al. [123] proposed that the overly simplistic dichotomy of M1/M2 macrophage phenotypes is inadequate, as it fails to capture the remarkable dynamism and plasticity macrophages exhibit across different tissues; the phenotype of macrophages is determined by many stimuli and cannot be adequately captured by a limited set of subtypes [124,125]. As shown in Figure 6, single-nucleus RNA sequencing (snRNA-seq) has revealed the presence of a distinct macrophage subpopulation in obesity-associated adipose tissue—namely, inflammatory and metabolically activated macrophages (IMAMs) characterized by high expression of *ATF4*, *PDIA3*, Acyl-CoA Synthetase Long Chain Family Member 4 (*ACSL4*), and C-C Motif Chemokine Ligand 2 (*CCL2*) (*ATF4^hi^PDIA3^hi^ACSL4^hi^CCL2^hi^*) [126]. These cells are significantly enriched in the visceral fat of obese patients, and their gene expression profiles indicate the simultaneous activation of pro-inflammatory pathways (such as TNF signaling and chemokine secretion) and metabolic dysregulation pathways (including fatty acid metabolism and insulin resistance). Pseudotime analysis further confirms that they reside at the initial stage of the differentiation trajectory of adipose tissue macrophages (ATMs), suggesting their critical pathogenic role in the progression of T2DM.

The risk of obesity-related metabolic diseases is closely linked to fat distribution. Excess accumulation of visceral fat (VAT) is directly associated with insulin resistance and T2DM, while the role of subcutaneous fat (SAT) is relatively complex [127,128]. Single-cell RNA sequencing analysis of VAT and SAT tissues from obese individuals revealed significant differences in the stromal vascular fraction (SVF) of the two fat depots, including differences in the subpopulations of immune cells, endothelial cells, and fibroblasts [129]. Visceral Progenitor with Mesothelial origin cells likely represent beige adipocyte progenitors with high mitochondrial activity and Uncoupling Protein 1 (UCP1) expression, potentially mitigating obesity-related metabolic dysfunction [130]. In parallel, a subpopulation of CD8+ T cells expressing metallothioneins (MT1F, MT1G, MT2A) has been linked to obesity, with expression levels correlating with BMI and a dysfunctional profile contributing to inflammation and insulin resistance [131,132]. Additionally, CD9+ metabolically active macrophages, characterized by high lipid metabolism gene expression, play a beneficial role in clearing dead adipocytes and maintaining tissue homeostasis in obesity [133]. These findings highlight the interplay between adipocyte progenitors, T cells, and macrophages in adipose tissue inflammation, providing insights into potential therapeutic targets for metabolic diseases.

## 7. Transformation Challenges from Data to Treatment

### 7.1. Research-Level Heterogeneity and Reproducibility

Single-cell omics has revolutionized our understanding of the cellular mechanisms underlying T2DM, yet major sources of discrepancy remain across studies. These variations arise from mechanistic heterogeneity, donor and population differences, and analytic variability.

Human studies consistently report features compatible with β-cell dedifferentiation, including the loss of mature identity genes (e.g., INS, MAFA, PDX1), accompanied by activation of stress-related and progenitor-like programs, although the initiating drivers of this process remain debated. Some studies emphasize ER stress and inflammatory signaling as upstream triggers of identity loss, whereas others prioritize disruption of core transcriptional networks as primary determinants [6,48].

Donor heterogeneity and population-specific variation further complicate interpretation. Differences in Body Mass Index (BMI), age, ethnicity, and sample sources such as surgical, cadaveric, and biobank specimens contribute to inconsistent transcriptional profiles across studies [134]. Such technical and cohort-level variability is compounded by population-level genetic and environmental diversity, which shapes disease susceptibility across populations [135]. Immune-related pathways often show population-specific differences that affect disease progression and treatment response, and limited sample diversity risks excluding underrepresented groups and worsening health disparities [136].

Batch effects are among the most persistent technical barriers to reproducibility. Variability introduced by platforms, reagents, and protocols can obscure true biological signals. Integration tools such as Seurat CCA and Harmony are widely used [137], yet over-correction can erase subtle biology while under-correction leaves bias unresolved [138]. Benchmark comparisons further reveal that preprocessing and integration choices strongly influence downstream results, affecting gene-expression variance, cluster resolution, and inferred cell states [73,139]. Table 2 summarizes representative computational frameworks across key analytical stages, highlighting their scalability, interpretability, and level of biological validation, as well as common pitfalls such as overcorrection and the generation of false-positive intermediate states.

Collectively, mechanistic ambiguity, cohort heterogeneity, and analytic inconsistency limit the reproducibility of findings and pose challenges for biological interpretation and clinical translation.

### 7.2. Clinical Transformation from Research to Application

Building upon the mechanistic insights discussed in previous sections, multi-omics discoveries provide a direct pathway from molecular characterization to clinically meaningful applications. Integrative analyses combining genetics and chromatin accessibility have delineated distinct β-cell subtypes whose transcriptional programs and risk-variant enrichment mark them as potential biomarkers of insulin-secretory decline [93]. Furthermore, the mechanistic understanding derived from single-cell data is guiding the optimization of cell-based therapies, including the generation of functional, transplantable stem-cell-derived β-like cells (SC-β-cells). Recent multi-omics analyses of human SC-islets have identified chromatin and transcriptional discrepancies relative to primary β-cells, offering insights to refine differentiation protocols and minimize off-target non-endocrine populations [142].

Despite these advances, practical translation remains constrained by systemic and technical barriers. The high cost of sequencing platforms and reagents presents a primary barrier to widespread clinical adoption, particularly in resource-limited settings [143]. In addition, the underrepresentation of diverse ancestral and geographic populations in publicly available single-cell datasets risks introducing algorithmic bias and reducing the generalizability of derived biomarkers [144]. To mitigate these issues, large-scale initiatives such as the Human Pancreas Analysis Program (HPAP) are expanding multi-ethnic donor representation and establishing harmonized data-integration standards [145].

Although no published study in T2DM has yet achieved a fully validated, decision-grade workflow in which single-cell data directly guide molecular subtyping or therapy selection, translational progress is accelerating. A recent study by Craig-Schapiro et al. established a vascular single-cell atlas of the human pancreas, revealing distinct endothelial subtypes and signaling disruptions linked to diabetes-associated microvascular dysfunction [146]. These insights connect single-cell discovery to clinically relevant mechanisms, offering new directions for vascular biomarker identification and therapeutic targeting. Collectively, such work illustrates how high-resolution cellular data are beginning to inform precision-medicine strategies and bridge the gap between mechanistic understanding and clinical application in diabetes care.

### 7.3. Future Outlook

The next phase of single-cell research in T2DM will require a transition from descriptive atlases to predictive and mechanistic frameworks. Enhancing the interpretability of computational models is a crucial step toward this goal. Moving beyond “black-box” algorithms through Explainable Artificial Intelligence (XAI) can uncover the regulatory logic of cellular transitions and improve biological interpretability, increasing translational value for clinical applications [147]. To establish causality rather than correlation, the integration of single-cell omics with high-throughput functional genomics such as CRISPR-based perturbation platforms like Perturb-seq will enable systematic testing of candidate regulators and accelerate the identification of disease-relevant pathways [148].

Capturing the dynamic and spatial complexity of metabolic tissues represents another critical frontier. Spatial-omics approaches now allow in situ mapping of cell–cell interactions and microenvironmental cues within pancreatic and adipose architectures, providing a contextual understanding of disease microanatomy [149]. Integrating such spatial and temporal dimensions will transform static single-cell measurements into clinically informative predictors of disease progression and treatment response.

Ensuring that these technological advances translate equitably into clinical practice remains a priority. Building diverse and inclusive reference atlases across ancestral and environmental backgrounds is essential to prevent algorithmic bias and ensure the generalisability of biomarkers [150]. Together, these efforts will advance single-cell multi-omics from a discovery-driven discipline to an actionable foundation for precision medicine in T2DM.

## 8. Conclusions

The rapid development of single-cell multi-omics technologies has revolutionized our understanding of the complex mechanisms underlying T2DM. These approaches have provided unprecedented insights into the cellular heterogeneity of pancreatic islets, adipose tissue inflammation, and disrupted intercellular communication, uncovering critical subpopulations and regulatory networks involved in disease progression. Computational advancements, such as batch effect correction, data denoising, multi-modal integration and machine learning, have further enhanced the ability to interpret these complex datasets, paving the way for discovering novel biomarkers and therapeutic targets.

Despite these advancements, significant challenges remain. Technical noise, data sparsity, and the difficulties of integrating multi-platform data continue to hinder the accurate interpretation of single-cell analyses. Additionally, cross-study integration is complicated by batch effects, requiring a delicate balance between removing technical variability and preserving true biological signals. A further limitation lies in the reliance on computational predictions, which often lack experimental validation, as well as the limited diversity of study populations, which restricts the generalizability of findings and risks exacerbating health disparities in precision medicine.

Future research must prioritize developing robust tools for data processing, improving the biological interpretability of computational models, and expanding the diversity of single-cell atlases. Efforts should also focus on ensuring that findings are experimentally validated and translated into clinically actionable insights. While challenges remain, single-cell multi-omics holds immense potential to unravel the complex biology of T2DM and advance precision medicine, bridging the gap between basic research and clinical application.

## Figures and Tables

**Table 1:** Performance comparison of data preprocessing algorithms.

Algorithms	Function	Key Principles	Limitations	Impact on Biological Interpretation
GRM [62]	Normalization	Use spike-in ERCC molecules to fit a gamma regression model between sequencing reads and RNA concentrations	Depends on spike-ins; less suitable for non-UMI data	Spike-in-based assumptions can bias low-abundance genes and distort DE analysis
BASiCS [63]	Normalization	Apply a unified Bayesian hierarchical framework to concurrently assess both the residual technical noise and the biological variability among cells	Require spike-in data for noise modeling, potentially biasing low-expression genes and limiting applicability to non-spike-in datasets	Over-reliance on spike-I ns may shrink true cell-to-cell variance
Scran [46]	Normalization	Group cells and deconvolve pooling size factors to address sparsity	Require careful cell clustering and may need additional batch correction	Mis-grouped cells bias normalization, reducing power to detect rare-cell markers
SCnorm [64]	Normalization	Use quantile regression to estimate gene-specific count-depth relationships	Require assumptions about gene grouping	Inaccurate grouping distorts depth corrections and alters downstream analysis
SCTransform [47]	Normalization	Regularize negative binomial regression with depth covariates and Pearson residuals	Not suitable for highly heterogeneous data; requires cross-gene parameter pooling to avoid overfitting	Strong regularization can compress biological variability though improves integration consistency
QUMI [65]	Normalization	Transform read counts to Poisson-log normal distributed quasi-UMI by quantile normalization to remove PCR amplification bias and simulate the distribution characteristics of the true UMI counts	Shape parameters need to be preset, and the difference in capture efficiency or gene length deviation cannot be completely eliminated	Inaccurate parameterization alters gene-ranking and inferred regulatory programs
ZIFA [66]	Recover dropout events	Extend factor analysis with a zero-inflation layer that models dropout events in scRNA-seq data via an exponential decay of dropout probability with latent expression levels	High computational complexity and assumptions that zeros stem purely from technical dropouts may overlook true biological silencing	May over-impute and create false intermediate states if biological zeros misclassified
CIDR [49]	Recover dropout events	Mitigate dropout effects by implicitly imputing missing gene expression values using a weighted mean based on estimated dropout probabilities	Unable to distinguish true low expression from technical dropout, and rely on predefined assumptions about the dropout probability relationship	Risk of artificial co-expression and blurred cluster boundaries
MAGIC [51]	Recover dropout events	Use data diffusion on a cell similarity graph to propagate gene expression information between similar cells	Over-smooth biologically relevant high-frequency variation and assume low-dimensional manifold structure	Oversmoothing merges distinct cell types and generates false “transition” states
DCA [50]	Recover dropout events	A deep count autoencoder network (DCA) to denoise scRNA-seq datasets; captures nonlinear gene-gene dependencies using a negative binomial noise model	Lead to overimputation in case of inadequate hyperparameter choices such as too low-dimensional bottleneck layer and hence data manifold	Over-imputation compresses variance and can invent pseudo-correlations
DeepImpute [67]	Recover dropout events	A deep neural network-based imputation algorithm that uses dropout layers and loss functions to learn patterns in the data	The model fitting step uses most of the computational resources and time	Enhances clustering coherence but may synthesize false co-expression
ScMultiGAN [68]	Recover dropout events	Employ a two-stage training process and utilizes multiple collaborative generative adversarial networks (GANs) to achieve cell-specific imputation	Requires significant computational resources and training time	GAN-based smoothing may create biologically implausible uniformity; needs external validation
MNN [56]	Correct batch effect	Match mutual nearest neighbors across batches to estimate and remove technical expression differences	Each batch contains at least one shared cell population with another batch	Over-correction merges distinct lineages, masking real biological differences
BBKNN [69]	Correct batch effect	Construct a batch-balanced k-nearest neighbor graph by identifying neighbors within each batch independently and merging them	Limit performance when cell type distributions or technical variations are highly uneven	Equalizing batch neighbor counts can blur subcluster boundaries
Harmony [57]	Correct batch effect	Follow dimensionality reduction via PCA, iteratively refine the alignment between cell clustering and batch distributions by employing soft clustering and localized linear corrections to mitigate batch effects	Overcorrection in batch effect removal may lead to the erasure of authentic biological differences	Excess alignment erases disease-specific signals
Scanorama [70]	Correct batch effect	Use an approximate nearest neighbor search based on hyperplane locality sensitive hashing and random projection trees	Inadvertently remove or blur genuine biological differences between batches	Misalignment possible when shared cell types are scarce
DESC [71]	Correct batch effect	Use deep learning with iterative optimization of a clustering objective function, leveraging autoencoders and soft cluster assignments to remove batch effects		Embedding instability can fragment continuous trajectories
scDML [72]	Correct batch effect	Leverage prior clustering information and intra-/inter-batch nearest neighbors within a triplet-based deep metric learning framework to simultaneously remove batch effects	Cannot be applied to datasets with differential structures; it solely creates integrated low-dimensional embeddings and does not provide corrected gene expression values	Gene-level interpretation limited; embeddings alone may hide subtle regulation

**Table 2:** Benchmark overview of computational methods for single-cell multi-omics.

Category	Representative Methods	Scalability	Interpretability	Biological Validation	Remarks
Batch correction & integration	Seurat (CCA), Harmony, LIGER, MOFA+	High	Moderate (latent features difficult to interpret)	Widely used; validated in human islet datasets	Balances accuracy and efficiency; risk of over-correction [57,77]
Dropout recovery	MAGIC, DCA, DeepImpute	Moderate	Moderate—Low	Partial validation in benchmarking datasets	Improves signal quality but may introduce artifacts or false-positive intermediate states [140]
Multi-modal data fusion	totalVI, scConfluence, MaxFuse	High	Moderate	totalVI and MaxFuse validated on PBMC and pancreas datasets	Enables cross-omics interpretation; computationally intensive [141]
Trajectory inference	Monocle, Slingshot	High	High	Extensively validated in β-cell and developmental datasets	Generates well-interpretable biological trajectories [30]
Cell–cell communication	CellPhoneDB, CellChat	Moderate	High	Experimentally confirmed in islet–immune interaction studies	Allows mechanistic inference at cell-type level; depends on curated ligand–receptor databases [9]
Machine-learning-based regulatory modeling	regX, XGBoost-based classifiers, deep learning models	High	Variable (often low)	Models validated for β-cell dysfunction	Offer powerful prediction but require improved interpretability and transparent feature attribution [87]

## Data Availability

No new data were created or analyzed in this study. Data sharing is not applicable to this article.

## Abbreviations

T2DM	Type 2 diabetes mellitus
INS	insulin
scRNA-seq	single-cell RNA sequencing
scATAC-seq	single-cell ATAC-seq
HNF1A	Hepatocyte Nuclear Factor 1 Alpha
HNF4A	Hepatocyte Nuclear Factor 4 Alpha
BBKNN	Batch Balanced K-Nearest Neighbors
WNN	Weighted Nearest Neighbor
MOFA+	Multi-Omics Factor Analysis v2
RFX6	Regulatory Factor X6
TNF	Tumor Necrosis Factor
UMAP	Uniform Manifold Approximation and Projection
PCA	Principal Component Analysis
LIGER	Linked Inference of Genomic Experimental Relationships
totalVI	Total Variational Inference
t-SNE	t-distributed Stochastic Neighbor Embedding
INS-IGF2	insulin–insulin-like growth factor 2
CHGA	chromogranin A
SCG5	secretogranin V
ER	endoplasmic reticulum
MAPK	Mitogen-Activated Protein Kinase
APCs	Adipose precursor cells
CD55	Decay-Accelerating Factor
CD9	Cluster of Differentiation 9
ICAM1	Intercellular Adhesion Molecule 1
CD142	Tissue Factor-positive
HbA1c	glycated hemoglobin
MDK	midkine
PEDF	pigment epithelium-derived factor
PROM1	Prominin-1
NFIB	Nuclear Factor I B
S100A11	S100 Calcium-Binding Protein A11
S100A6	S100 Calcium-Binding Protein A6
NKX2-2	NK2 Homeobox 2
G6PC2	Glucose-6-Phosphatase Catalytic Subunit 2
CAPS	Calcyphosine
PRDX2	Peroxiredoxin 2
CPM	counts per million
TPM	transcripts per million
CCA	Canonical Correlation Analysis
rIOT	regularized Inverse Optimal Transport
MDT	Mosaic Data Topology
GNNs	graph neural networks
CyTOF	Cytometry by Time-of-Flight
CIDR	Clustering through Imputation and Dimensionality Reduction
UINMF	Unshared Integrative Non-negative Matrix Factorization
U-Net	U-shaped Convolutional Neural Network
MACS2	Model-based Analysis of ChIP-Seq v2
DCA	Deep Count Autoencoder
MAGIC	Markov Affinity-based Graph Imputation of Cells
JNK	c-Jun N-terminal Kinase
NF-κB	Nuclear Factor kappa-light-chain-enhancer of activated B cells
HNF	Hepatocyte Nuclear Factor
GLIS3	GLIS Family Zinc Finger 3
RORA	RAR-Related Orphan Receptor Alpha
hESCs	human embryonic stem cells
SC-β-cells	stem cell-derived β-like cells
T	Brachyury
SOX17	SRY-Box Transcription Factor 17
PDX1	Pancreatic and Duodenal Homeobox 1
NEUROG3	Neurogenin 3
IAPP	Islet Amyloid Polypeptide
PAX4	Paired Box 4
TCF7L2	Transcription Factor 7 Like 2
WGCNA	weighted gene co-expression network analysis
GCK	Glucokinase
ND	non-diabetic
INSR	insulin receptor
GCG	glucagon
GCGR	glucagon receptor
SST	somatostatin
SSTR	somatostatin receptor
C5AR1	Complement Component 5a Receptor 1
RPS19	Ribosomal Protein S19
PBMCs	peripheral blood mononuclear cells
CD30	Cluster of Differentiation 30
CD48	Cluster of Differentiation 48
TGF-β	Transforming Growth Factor Beta
IFN-γ	Interferon Gamma
snRNA-seq	single-nucleus RNA sequencing
IMAMs	metabolically activated macrophages
ATF4	Activating Transcription Factor 4
PDIA3	Protein Disulfide Isomerase Family A Member 3
ACSL4	Acyl-CoA Synthetase Long Chain Family Member 4
CCL2	C-C Motif Chemokine Ligand 2
ATMs	adipose tissue macrophages
VAT	visceral fat
SAT	subcutaneous fat
SVF	stromal vascular fraction
UCP1	Uncoupling Protein 1
HPAP	Human Pancreas Analysis Program
BMI	Body Mass Index
XAI	Explainable Artificial Intelligence
